# Artificial Intelligence Understands Peptide Observability and Assists With Absolute Protein Quantification

**DOI:** 10.3389/fpls.2018.01559

**Published:** 2018-11-13

**Authors:** David Zimmer, Kevin Schneider, Frederik Sommer, Michael Schroda, Timo Mühlhaus

**Affiliations:** ^1^Computational Systems Biology TU Kaiserslautern, Kaiserslautern, Germany; ^2^Molekulare Biotechnologie & Systembiologie TU Kaiserslautern, Kaiserslautern, Germany

**Keywords:** mass spectrometry, deep learning, machine learning, proteotypic peptide, peptide observability, absolute quantification

## Abstract

Targeted mass spectrometry has become the method of choice to gain absolute quantification information of high quality, which is essential for a quantitative understanding of biological systems. However, the design of absolute protein quantification assays remains challenging due to variations in peptide observability and incomplete knowledge about factors influencing peptide detectability. Here, we present a deep learning algorithm for peptide detectability prediction, d::pPop, which allows the informed selection of synthetic proteotypic peptides for the successful design of targeted proteomics quantification assays. The deep neural network is able to learn a regression model that relates the physicochemical properties of a peptide to its ion intensity detected by mass spectrometry. The approach makes use of experimentally detected deviations from the assumed equimolar abundance of all peptides derived from a given protein. Trained on extensive proteomics datasets, d::pPop's plant and non-plant specific models can predict the quality of proteotypic peptides for not yet experimentally identified proteins. Interrogating the deep neural network after learning from ~76,000 peptides per model organism allows to investigate the impact of different physicochemical properties on the observability of a peptide, thus providing insights into peptide observability as a multifaceted process. Empirical evaluation with rank accuracy metrics showed that our prediction approach outperforms existing algorithms. We circumvent the delicate step of selecting positive and negative training sets and at the same time also more closely reflect the need for selecting the top most promising peptides for targeting a protein of interest. Further, we used an artificial QconCAT protein to experimentally validate the observability prediction. Our proteotypic peptide prediction approach not only facilitates the design of absolute protein quantification assays via a user-friendly web interface but also enables the selection of proteotypic peptides for not yet observed proteins, hence rendering the tool especially useful for plant research.

## Introduction

System-wide studies to identify and quantify the protein components are key to understand the complex cellular dynamics in response to system perturbations. Therefore, mass spectrometry-based proteomics approaches have become an integral element for biological research. While relative quantification methods are suited to generate accurate and comprehensive data series on changes of a cell's protein content, targeted proteomics approaches can provide absolute quantification information, which are essential for biochemical system simulations during perturbations (Kuster et al., 2005). To quantify selected proteins of interest, a known amount of synthetic proteotypic peptides (PTPs) that mimic peptides produced by the proteolytic cleavage of target analyte proteins, are spiked into a cell extract. Either the synthetic peptides or the proteins in the extract are labeled with stable isotopes, thus leading to light and heavy peptide pairs. After ionization, these pairs can be separated and quantified by mass spectrometry, with the synthetic peptide serving as calibrator (Barnidge et al., 2003; Gerber et al., 2003).

However, the selection of PTPs suited for the absolute quantification of the target proteins remains challenging and cumbersome. The difficulties are due to the large variation of observability of peptides from the same protein with knowledge on the factors influencing the detectability being fragmentary. Two strategies how to select appropriate surrogate peptides for absolute quantification are commonly present in the literature: the first is a selection process based on expert rules. Here PTPs are chosen from previously performed mass spectrometry experiments dedicated to identify the proteins of interest, or this information is taken from public repositories like PeptideAtlas (Desiere et al., 2006), Pride (Martens et al., 2005), SRMAtlas (Picotti et al., 2008; Kusebauch et al., 2016), Panorama (Sharma et al., 2014), and SwathAtlas (Rosenberger et al., 2014). Experimentally gained information on the presence of the peptides is then combined with rule-based criteria like the existence of possible modifications, missed cleavages, and the presence or absence of certain amino acids to select suitable PTPs (Bereman et al., 2012; Mohammed et al., 2014; Scott et al., 2016). However, if experimental data is incomplete, as is the case in plant proteomics research, the observability of many peptides cannot be assessed.

For yet unidentified proteins, the second selection strategy relies on the accurate prediction of the observability of a peptide for targeted quantification. It could be shown that the computationally assisted assay design is more cost- and time-efficient compared to unautomated expert design approaches (Picotti et al., 2010). ChemScore is an implementation of such a decision-based strategy in the context of MALDI-MS (Parker, 2002). In the past, several algorithms have been proposed that challenge PTP prediction with different machine learning methods. Those methods are general-purpose approaches to learn relationships from data and follow the canonical workflow: (i) data collection and defining a training set, (ii) numerical feature extraction, (iii) fitting a predictive model, and (iv) model evaluation. Following this generic process, the algorithms available for PTP prediction differ regarding the definition of the learning problem and therefore in the realization of the respective steps regarding the canonical workflow. Most of the approaches construct a binary classification problem and sort the peptides into the two categories of observable (“flyers”) and non-observable (“non-flyers”) peptides. This idea was shown to be applicable when peptide detectability was first described in the context of LC-MS by Tang et al. who proposed a proof-of-concept study, training an ensemble of small neural networks serving as the first machine learning-based predictor of peptide detectability (Tang et al., 2006). PeptideSieve (Mallick et al., 2007) applies a Gaussian mixture model and CONSeQuence (Eyers et al., 2011) a combination of different machine learning techniques on peptides that have been detected in at least 50% of the times that their parent protein was observed in high quality yeast data sets. STEPP (Webb-Robertson et al., 2008) uses support vector machines (SVM) to build a model from non-identified and identified peptides previously reported in high quality accurate mass and elution time (AMT) proteomics studies. ESPPredictor (Fusaro et al., 2009) uses Random Forest to classify peptides according to their threshold-based high or low signal peak intensity at the precursor ion level.

Here we present d::pPop, a deep learning algorithm for peptide detectability prediction, that circumvents the binary classification and therefore the critical distinction between peptides that have not been observed experimentally, yet, and those that truly are non-observable. Theoretically, proteotypic peptides deriving from the same protein should all be present in equimolar abundances. Experimentally observed deviations from this can only be explained by differences in peptide observability, that depends on ionization efficiency, variability in signal acquisition, digestion efficiency, and the occurrence of post-translational modifications. Therefore, we rank the peptides within the same protein according to their measured abundance and convert the problem to a “learning to rank” problem. Classical learning methods like SVMLight, RankNet, or LambdaMart, designed to solve ranking problems, are usually used in information retrieval and web searches and were first applied to the peptide ranking problem by the PeptideRank algorithm (Qeli et al., 2014). Here we designed a deep learning network structure dedicated to the specific properties of the peptide ranking problem. d::pPop accounts for the essential difference between peptide ranking compared to ranking web sites, that is the relation between the query peptide's relative abundance within its parent protein is statistically linked to its physicochemical features and is continuous. Therefore, we consider the proteomics workflow as a special ranking process where the relative deviation from the maximal detectability of a given peptide can be explained by its physicochemical properties. By learning from ~150,000 peptide sequences from plant and non-plant organisms, we can optimally capture the difference in peptide observability. In a systematic evaluation that reflects the peptide selection principle for targeted proteomics, our approach outperforms previously published PTP prediction algorithms. Further, d::pPop's artificial intelligence can be interrogated after learning from the measurement data about factors that determine peptide observability.

## Materials and methods

### Datasets and data processing

The establishment of our method required the assembly of large shotgun proteomic data sets for both model organisms, baker's yeast (*Saccharomyces cerevisiae*) and *Chlamydomonas reinhardtii* (*C. reinhardtii*). To achieve an extensive coverage of observable and quantifiable peptides, we blended proteomics data from several studies into two distinct assemblies, one for each model organism. For both assemblies, peptide spectrum matches (PSMs) of fully tryptically digested peptides with a MaxQuant posterior error probability of at most 0.01 were considered. The resulting lists were subsequently filtered for PTPs (Qeli and Ahrens, 2010). As an estimator for peptide observability, the areas of the extracted ion chromatograms (XIC) were chosen and peptides were ranked accordingly, with a lower rank indicating a higher abundance. This vast collection of peptides was then expanded by including peptides that have not been quantified, i.e., which had an XIC value of zero. The yeast data sets were downloaded from the PRIDE repository (IDs: PXD000409, PXD002694, PXD004028, PXD004028, PXD005041, and PXD005795). All data sets from *C. reinhardtii* were combined from previous proteome-wide studies (Mühlhaus et al., 2011; Hemme et al., 2014; Mettler et al., 2014; Schmollinger et al., 2014; Werth et al., 2017). Both data sets were subsequently filtered for single occurrence of all proteins. If a protein was measured in more than one study, we kept the protein with most identified peptides to maximize the information in the data sets (Supplemental Data Sheet 1). Subsequently, we randomly removed 20% of the proteins in each assembly to use them as validation-data-sets to control for sufficient generalization of trained deep neural networks (DNNs). Thus, we obtained two training datasets consisting of 2,652 yeast and 2,732 *C. reinhardtii* proteins and two validation datasets consisting of 664 yeast and 685 *C. reinhardtii* proteins. An additional test dataset for the plant model was constructed accordingly, using *Arabidopsis thaliana* data downloaded from the PRIDE repository (IDs: PXD006257) containing 1,074 proteins.

### Learning to rank algorithms

Many modern applications such as document retrieval, object rating or product recommendation rely on the accurate ranking of candidate entities, which led to the development of various machine learning algorithms targeting the “learning to rank” problem. Besides the mentioned applications, also the prediction of observable peptides can be formulated as a ranking problem. In proteomics experiments, a set of m proteins Pr = {pr^1^, pr^2^,…, pr^m^} is thought to be present in the possibility space. Due to the nature of shotgun proteomics approaches, each protein pr^i^ is indirectly measured by a set of n possible peptides P^i^ = {p${}_{1}^{\text{i}}$, p${}_{2}^{\text{i}}$,…, p${}_{\text{n}}^{\text{i}}$}, where n results as a direct consequence of protein sequence and selected digestion parameters. Moreover, each set of peptides P^i^ is assigned to a set of n scores S^i^ = {s${}_{1}^{\text{i}}$, s${}_{2}^{\text{i}}$,…, s${}_{\text{n}}^{\text{i}}$}, where s${}_{\text{j}}^{\text{i}}$ reflects the observability of p${}_{\text{j}}^{\text{i}}$ with respect to protein pr^i^. Choosing the score s${}_{\text{j}}^{\text{i}}$ is typically open to the researcher and can thus take different shapes, like the logarithmic transformation of spectral counts (Qeli et al., 2014) or, in our case, the relative XIC intensity, obtained by grouping training set peptides by their protein identifier and subsequent normalization by the most abundant peptide to correct for differences in protein abundance.

Subsequently, each peptide sequence is processed using the BioFSharp framework (available at: https://github.com/CSBiology/BioFSharp) and converted into a feature vector with 45 entries which represents a numerical footprint of physicochemical peptide properties. The selected set of peptide features is based on an initial library of 574 features of the AAindex1 (Kawashima, 2000). From this library, amino acid frequencies and ten general properties were chosen: molecular weight, isoelectric point, peptide length, net charge, positively charged residues, negatively charged residues, relative frequency of polar amino acids, and relative frequency of hydrophobic amino acids, and relative frequency of negatively charged amino acids. This set is extended by subsets of features retrieved from the AAindex1 (Kawashima, 2000), sampled, and further optimized to contain minimal redundancy while retaining maximum relevance. This optimization included pairwise feature correlation followed by hierarchical clustering, minimum spanning tree analysis, and subsequent cluster-wise importance ranking (Qeli et al., 2014). Thereby, the list of features was extended by Activation Gibbs energy of unfolding at pH 9.0 (Yutani et al., 1987), amino acid composition of MEM of single-spanning proteins (Nakashima and Nishikawa, 1992), principal component II (Sneath, 1966), hydrophobicity index (Wolfenden et al., 1981), the Chou-Fasman parameter of coil conformation (Charton and Charton, 1983), average number of surrounding residues (Ponnuswamy et al., 1980), interior composition of amino acids in intracellular proteins of mesophiles (Fukuchi and Nishikawa, 2001), weights for coil at the window position of −3 (Qian and Sejnowski, 1988), helix formation parameters (delta delta G) (O'Neil and DeGrado, 1990), free energy in alpha-helical regions (Muñoz and Serrano, 1994), average relative fractional occurrence in EL (i) (Rackovsky and Scheraga, 1982), and composition of amino acids in extracellular proteins (Cedano et al., 1997). We further extended this list by the hydrophobicity index 2, the frequency of occurrence of missed tryptic cleavage sites in the peptide sequence, and C- and N-terminal digestion probabilities (Kawashima, 2000; Siepen et al., 2007).

### Deep peptide observability predictor (d::pPop)

**Figure 1:**
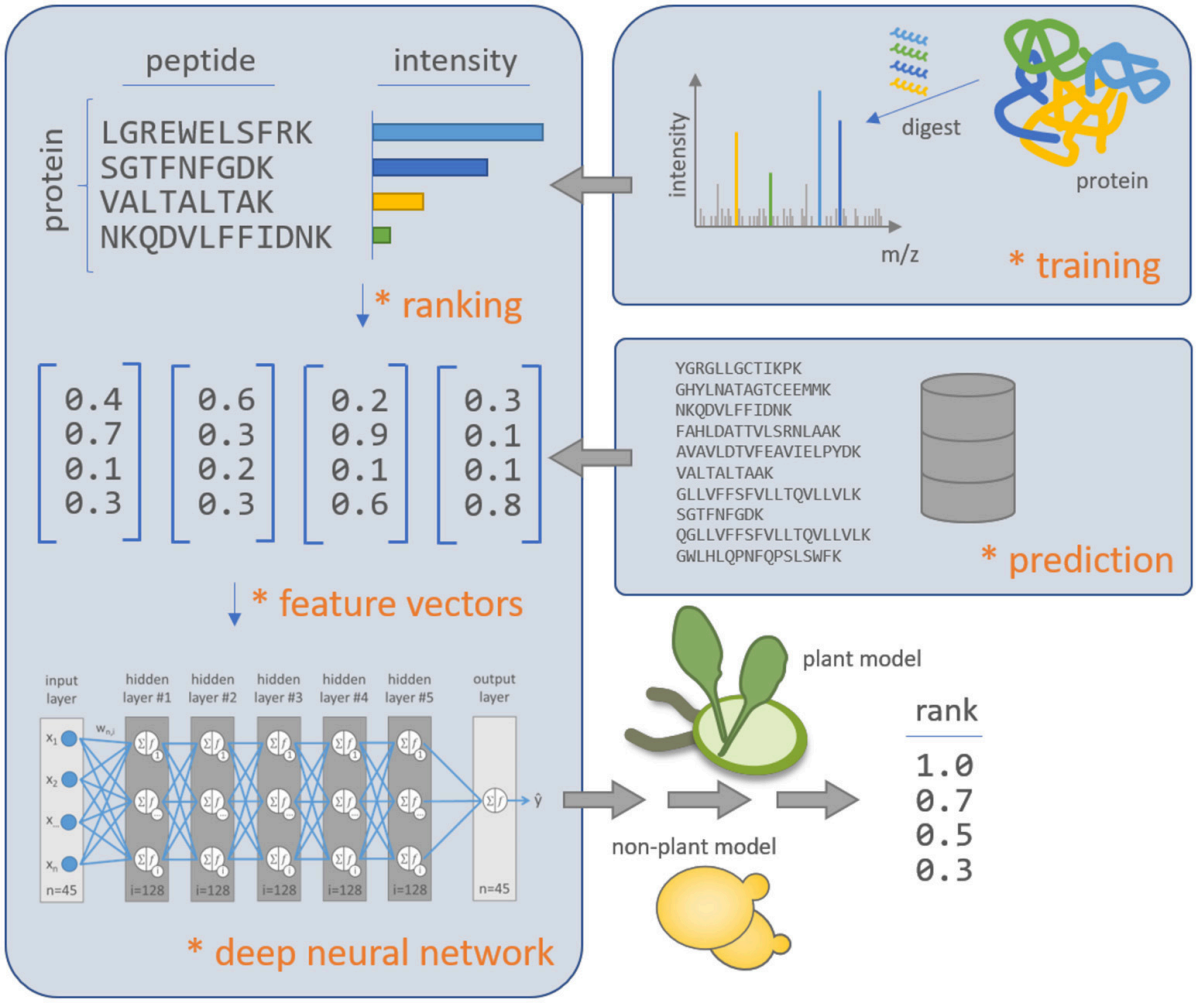
Schematic overview of the deep learning approach d::pPop to predict the rank of peptide observability within plant and non-plant specific query proteins. The algorithm is based on deep neural networks and is trained on experimentally observed proteins with all PTPs protein-wise normalized to the peptide with maximum intensity to match the assumption of equal molarity. The feature vectors are computed to represent the physicochemical properties of the peptide sequences. The deep neural network is able to learn a regression model that relates the physicochemical peptide properties to the difference in peptide intensities within a single protein in the proteomics workflow. The plant and non-plant specific models can predict the quality of PTPs for not yet experimentally identified proteins.

The fundament of the d::pPop algorithm is a deep fully connected feed forward neural network architecture implemented in F# using the Microsoft Cognitive Toolkit (CNTK, available at https://github.com/Microsoft/CNTK). The network architecture and training parameters were applied irrespective of the organism-specific data used for training. The basic network architecture consists of five dense layers with 128 nodes each (Figure 1). Neurons were modeled as rectified linear units (ReLUs), since it was shown that DNNs with ReLUs train several times faster and minimize the risk of problems with vanishing gradients (Krizhevsky et al., 2017). To reduce the risk of overfitting, we relied on the very efficient “dropout” technique, which at a probability of 0.2 sets the output of a ReLU to zero, thus eliminating its influence on the back propagation of the gradient during the training phase (Hinton et al., 2012). The networks were trained using a minibatch size of 3 for 10 epochs and a training set of 76,117 yeast peptides and 76,962 *C. reinhardtii* peptides. As a loss function, we relied on the squared error between predicted observability and normalized peptide intensity (see section on Learning to Rank Algorithms). The optimization of network weights was carried out using a stochastic gradient descent at a learning rate of 0.001.

### Evaluation of ranking accuracy

Since the efficient design of QconCAT proteins is tied to the selection of only a small set of peptides per protein of interest, it is favorable to use a metric that credits a high prediction accuracy on the top results, while being robust to permutations of lower ranking peptides. A measure that gained high popularity among the information retrieval community and fulfills the aforementioned requirements is the normalized discounted cumulative gain (nDCG) implemented as:

$$\begin{array}{l} nDCG@k=\;\frac{\sum_{k}^{i\;=\;1}\frac{\log_{2}s(p_{i})}{\log_{2}(1+i)}}{\sum_{k}^{i\;=\;1}\frac{\log_{2}s(q_{i})}{\log_{2}(1+i)}} \end{array}$$

Where @k determines until which position the ranking quality is examined, s resembles the relevance score (see section Learning to Rank Algorithms), and p_i_ and q_i_ being the predicted peptide at ith position and the observed peptide at ith position, respectively (Järvelin and Kekäläinen, 2002).

### Evaluation of feature importance

While the selection of features is driven by considerations of human experts, the analysis of their contribution to the activation potentials of the neural network nodes can give insights into the relevance learned by the network after being confronted with thousands of real-world examples. To do so, we analyzed the activation potentials of ReLUs present in the first hidden layer of our networks (Roy et al., 2015). For a feature i of a training example x the activation potential of a first hidden layer neuron j is calculated as:

$$\begin{array}{l} a_{ij}=\;w_{ji}x_{i}+\;b_{j} \end{array}$$

with w_j_ being the learned weight and b_j_ the learned bias. The average activation potential p_ij_ of one feature i can be estimated across M training examples using:

$$\begin{array}{l} p_{ij}=\;\frac{1}{M}\overset{M}{\underset{k=1}{\sum }}|a_{ij}^{k}| \end{array}$$

which can then be used to calculate the relative contribution c_ij_ of the ith input feature by:

$$\begin{array}{l} c_{ij}=\;\frac{a_{ij}}{\sum_{N}^{i=1}p_{ij}} \end{array}$$

Finally, summing across all first hidden layer neurons H leads to the net positive contribution c${}_{\text{i}}^{+}$ of one feature as:

$$\begin{array}{l} c_{i}^{+}=\overset{H}{\underset{j=1}{\sum }}f_{R}\left(c_{ij}\right) \end{array}$$

with f_R_ being the activation function of a ReLU:

$$\begin{array}{l} f_{R}\left(a_{j}\right)=\max\left(0,a_{j}\right) \end{array}$$

### QconCAT protein LC-MS/MS analysis

The photosynthesis QconCAT protein (PS-Qprot) used to evaluate the d::pPop PTP predictions was described by Hammel et al. (2018) in this issue. Here, 50 μg of total *C. reinhardtii* protein (corresponding to 3.87 × 10^6^ cells) were mixed with 0.25, 0.5, 2.5, and 5 μg ^15^N-labeled PS-Qprot, respectively. Samples were measured using a Triple-TOF 6600 (Sciex). LC-MS/MS run and sample preprocessing was carried out as described in Hammel et al. (2018). BioFSharp was used for the extraction of ion chromatograms and for the quantification of peak areas of heavy Q-peptides and light native peptides.

## Results

### Deep learning to rank workflow

The d::pPop algorithm presented here aims to be an all-in-one solution for the selection of PTPs to be used for absolute protein quantification. This is achieved by the combination of a feature extraction workflow developed using the BioFSharp toolkit with recently developed machine learning tools (Yu et al., 2014). The algorithm is based on the idea that peptide observability can be interpreted as a dependent variable that can be estimated by regression-based methods.

The whole workflow is explained best when focusing on the retrieval of PTPs for a protein of interest. (i) As a first step, d::pPop retrieves all tryptic peptides for a given protein from a user-provided database. (ii) This set of peptides is then classified (Qeli and Ahrens, 2010) and only peptides of class 1a, representing distinct peptides, are considered as PTPs and retained for further processing steps. (iii) Remaining peptides are analyzed and corresponding feature vectors are calculated. The selection of peptide features represented in the vectors includes the relative amino acid abundance as well as a set of ten general properties (e.g., molecular weight, isoelectric point). This set was further enlarged by a subset of properties present in the AAindex1 (Kawashima, 2000). This subset was created by an optimization procedure aiming to achieve minimal redundancy while retaining maximum relevance (Qeli et al., 2014). Each computed feature vector serves as a numerical physicochemical footprint of a peptide and can be viewed as a set of independent variables which, mapped by a function, provides us with an estimated peptide observability. (iv) Consequently, these feature vectors are then fed into a DNN assigning a predicted observability to each peptide based on its feature vectors. This observability is then normalized by the highest scoring peptide to provide the user with a result list that is ranked in descending order by score.

The formulation of peptide observability as a dependent variable fits the capabilities of DNNs, which are theoretically capable to approximate any bounded continuous function (Mitchell, 1997). After training [see section Deep Peptide Observability Predictor (d::pPop)], the neural network (with five hidden layers and 128 neurons each) is capable of using learned weights and biases, thereby capturing complex feature relationships present in the training data. These act as a multivariate function, thereby mapping computed feature vectors to peptide observabilities, which undergo further processing as described above. To account for putatively occurring organism-specific differences, we used two distinct training sets (see section Datasets and Data Processing) and incorporated both trained DNNs into d::pPop.

### Comparison of prediction performance

In a thorough empirical evaluation, we compared d::pPop with existing PTP predictors including the rule-based approach ChemScore (Parker, 2002) as well as machine learning-based approaches represented by PeptideSieve (Mallick et al., 2007), PeptideRank (Qeli et al., 2014), CONSequence (Eyers et al., 2011), and ESPPredictor (Fusaro et al., 2009). Since all used data sets contain quantitative information about each peptide, we can calculate the relative rank of each peptide within a given protein and use the nDCG as a suitable metric for performance evaluation. The nDCG metric credits a high prediction accuracy according to its relevance for ranking and ranges from zero to one, indicating random to correctly ranked top peptides according to observed abundance (see section Evaluation of Ranking Accuracy). This approach is well-suited for the aim of targeted proteomics, where a small number of representative peptides is selected to quantify a given target protein.

**Figure 2:**
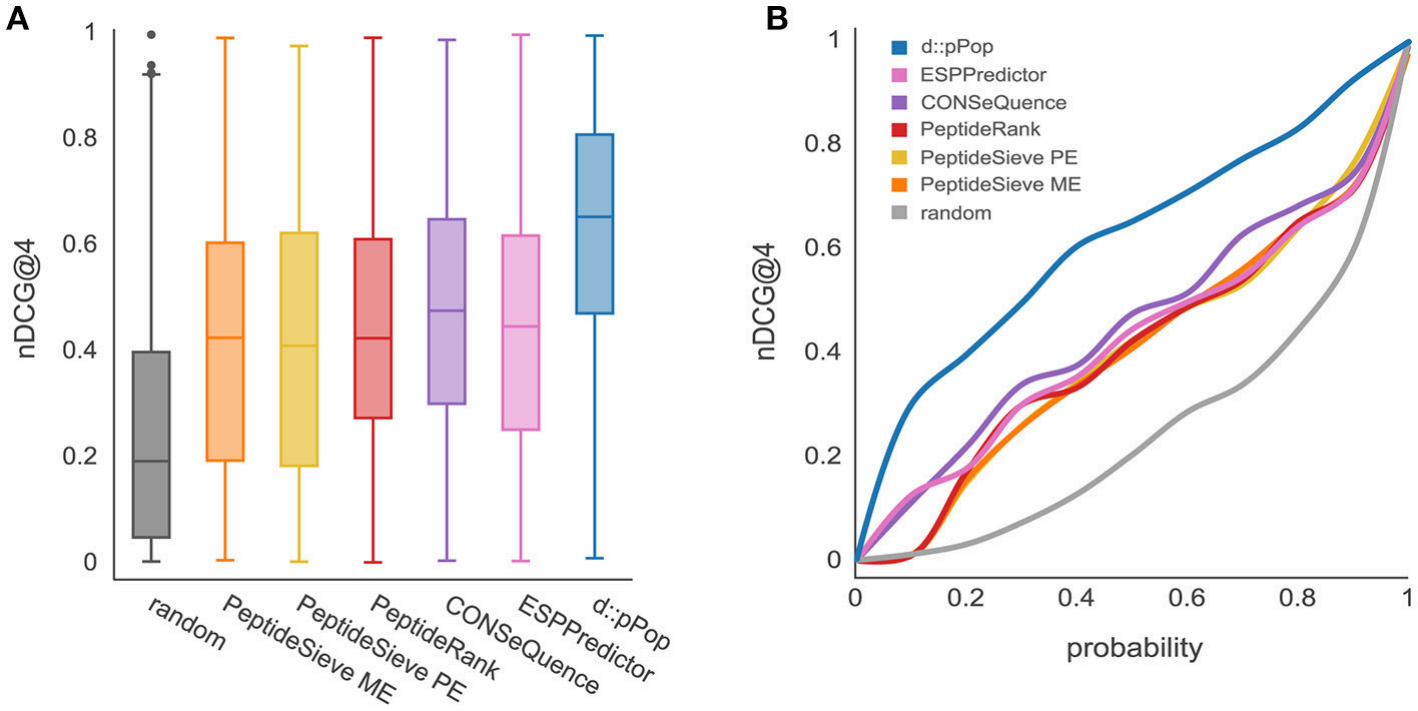
Prediction results using d::pPops non-plant model in comparison with common PTP predictors for the yeast proteome. The evaluation was performed using a yeast proteome data set consisting of 664 proteins. **(A)** According to the nDCG@4 as a measure of ranking accuracy shown with box plots for the different prediction results, all algorithms are consistently performing better than the randomized ranking of peptide queries. However, it can be observed that d::pPops ranking accuracy is higher in average compared to the other PTP predictors. **(B)** The corresponding cumulative distribution representation reflects the more accurate prediction by the line being closer to a constant nDCG@4 value of 1.

As most of the previously released PTP predictors have been trained on LC-MS/MS data sets from yeast, we first analyzed the performance of d::pPop that has been trained on 2,652 yeast proteins to predict the 664 remaining proteins of the yeast proteome. In this comparison, d::pPop achieved a higher median nDCG@4 score than the other predictors (Figure 2A). The corresponding cumulative distribution (Figure 2B blue line) indicates a more accurate prediction by being closer to a constant nDCG@4 value of 1 corresponding to an ideal ranking. The data demonstrate that d::pPop outperforms the other PTP predictors in a rank-based comparison.

### Plant species specificity

**Figure 3:**
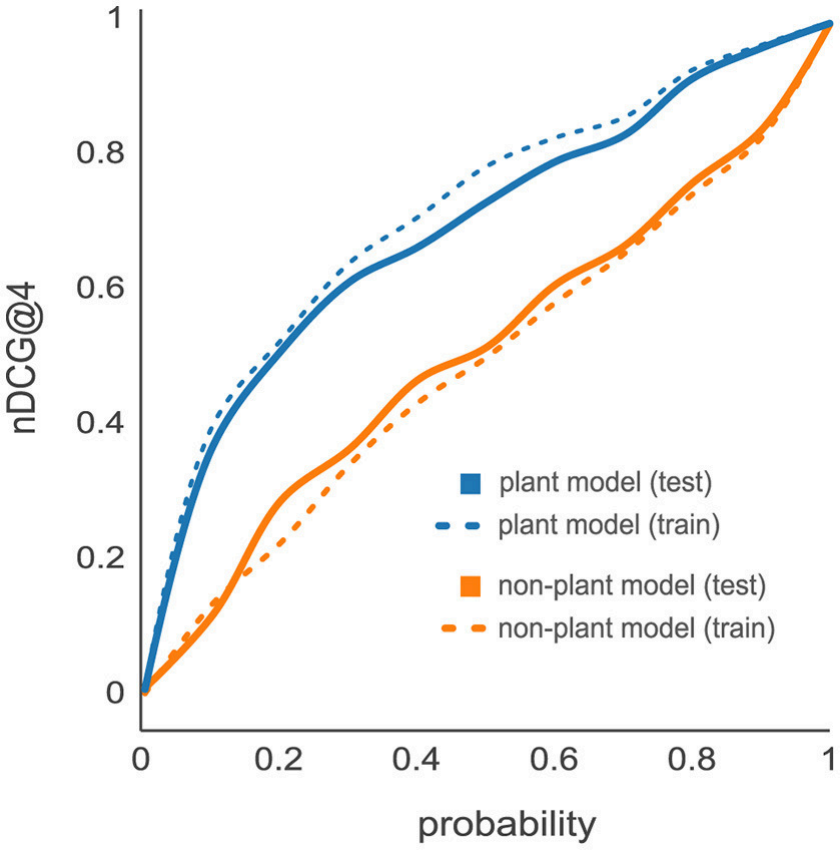
Effect of organism-specific models on PTP predictor accuracy. Comparison of d::pPop prediction results for a *Chlamydomonas reinhardtii* proteome data set using d::pPop's non-plant (orange lines) and plant (blue lines) model. The cumulative distributions of the nDCG@4 showed only a small difference between predictions on training (dashed lines) and test (solid lines) data sets. The data indicates that the models do not suffer from extensive overfitting since the performance does not differ substantially. However, it can be observed that the non-plant model generalizes imperfectly, which indicates that prediction is indeed organism-specific.

Previous studies suggest an organism-specific effect on the PTP predictive power caused by organism-dependent differences in amino acid frequencies and therefore reflected by the peptide properties (Webb-Robertson et al., 2008; Qeli et al., 2014). To analyze organism-specific effects, we compared the nDCG@4 cumulative distributions resulting from the d::pPop predictions on the *C. reinhardtii* proteome data set based on d::pPops non-plant (orange lines) and plant (blue lines) models. The non-plant model was trained on yeast, the plant model was trained on *C. reinhardtii* LC-MS/MS data sets. First, we evaluated the prediction accuracy on the training data set of 2,732 proteins (dashed lines) in comparison to the test data set consisting of 685 proteins (solid lines) (Figure 3). The small difference in performance between training and test data for the same model evidences that both d::pPop models do not suffer from overfitting. The comparison of how the two models predict peptide observability for the *C. reinhardtii* test data revealed a substantial difference in prediction accuracy. This is also true for the prediction of the *A. thaliana* test data set (Supplemental Figure 1). This indicates that prediction is organism-specific (Figure 3, solid lines).

**Figure 4:**
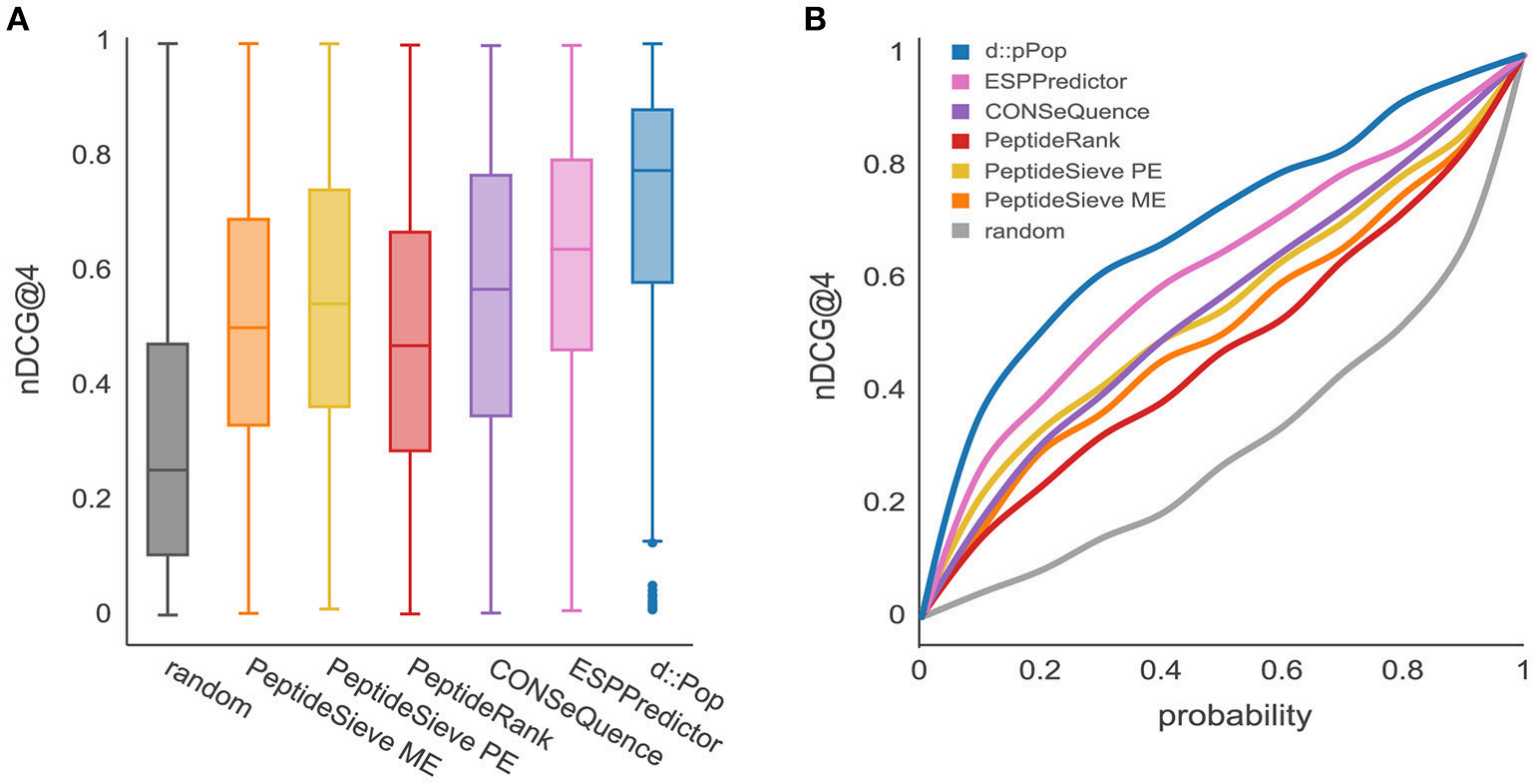
Prediction results for the *Chlamydomonas reinhardtii* proteome using d::pPop's plant model in comparison with common PTP predictors. The evaluation was performed using a *C. reinhardtii* proteome data set consisting of 685 proteins. **(A)** According to the nDCG@4 presented with box plots for the different prediction results, all algorithms showed a nDCG@4 that was consistently higher in average than the randomized ranking of peptide queries. However, it can be observed that d::pPops ranking accuracy is superior in comparison to existing PTP predictors. **(B)** The corresponding cumulative distribution representation reflected the more accurate prediction by the line being closer to a constant nDCG@4 value of 1.

The observed organism-specific effect calls for a comparison of d::pPop with other PTP predictors regarding their predictive power on the plant data set. Most predictors produced a higher median nDCG@4 score on the plant data than on the yeast data, thus corroborating the organism bias on the predictive power (Figure 4). Furthermore, as was true for the yeast data set, the median nDCG@4 scores reached by the other PTP predictors were below that reached by d::pPop. This suggests that d::pPop performs best in rank-based comparisons, thus recommending d::pPop for the prediction of peptide observability in plant proteomics.

### Experimental validation of d::pPop predictions

**Figure 5:**
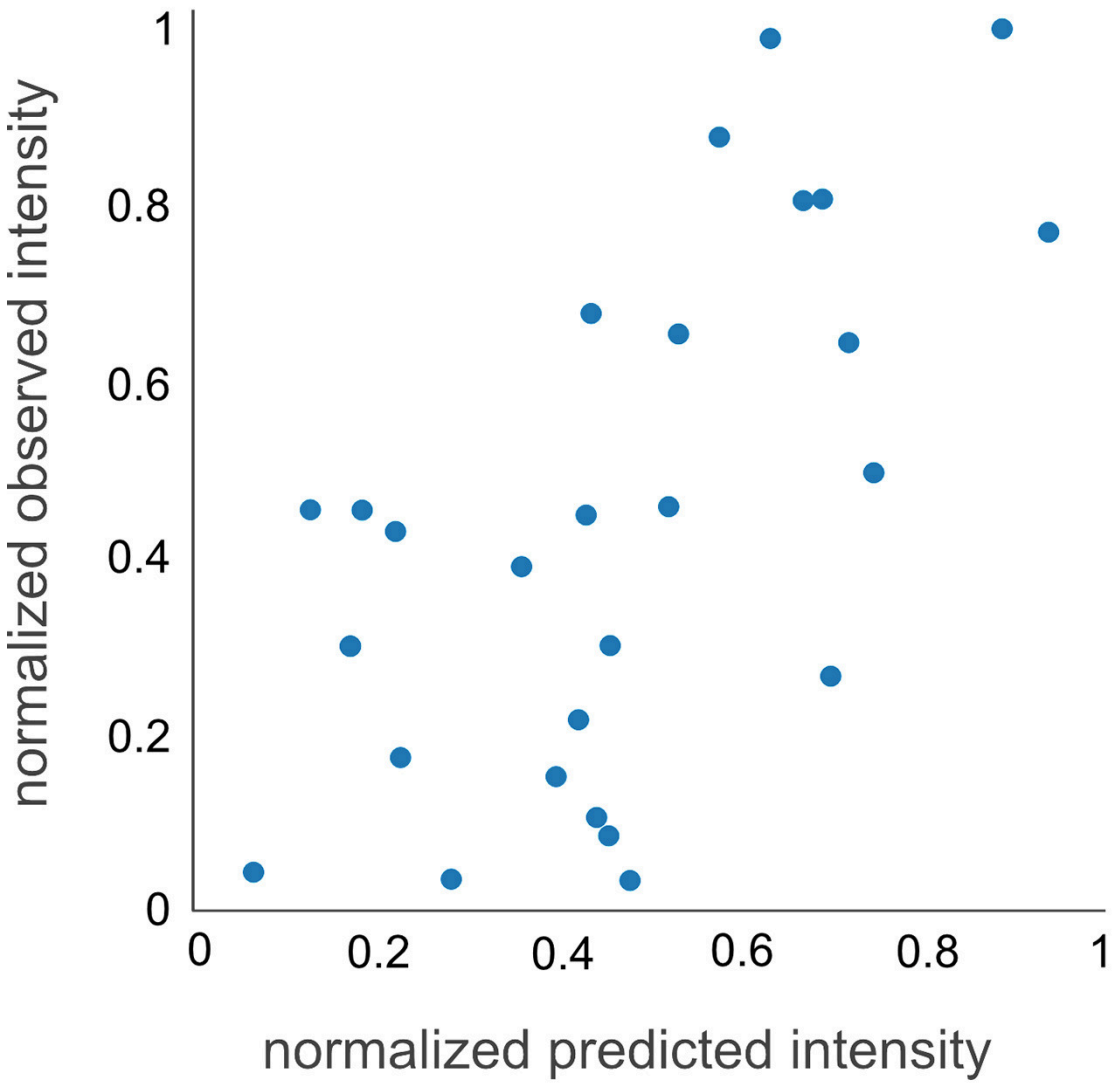
QconCAT observability prediction. The prediction of the normalized intensities of the Q-peptides is in solid agreement with the measured normalized intensities, showing a Pearson correlation coefficient of 0.62.

To experimentally test the observability prediction, we applied d::pPop to predict the observability of 32 peptides from a QconCAT protein that was tryptically digested and analyzed by LC-MS/MS. This QconCAT protein (PS-Qprot) targets ten soluble and membrane-intrinsic photosynthesis proteins in *C. reinhardtii* (Hammel et al., 2018). The measurement of all PS-Qprot peptides rendered an artificial scenario that could not be accounted for in the training dataset. A Pearson correlation coefficient of 0.62 suggested a decent agreement between measured and predicted normalized peptide abundances (Figure 5). In contrast to the evaluation based on the nDCG@4 score, this result indicates that the prediction of the final rank is possible and that the regression on the relative peptide abundance is according to the measurement.

### D::pPop selects optimal signature peptides for targeted proteomics assays

**Figure 6:**
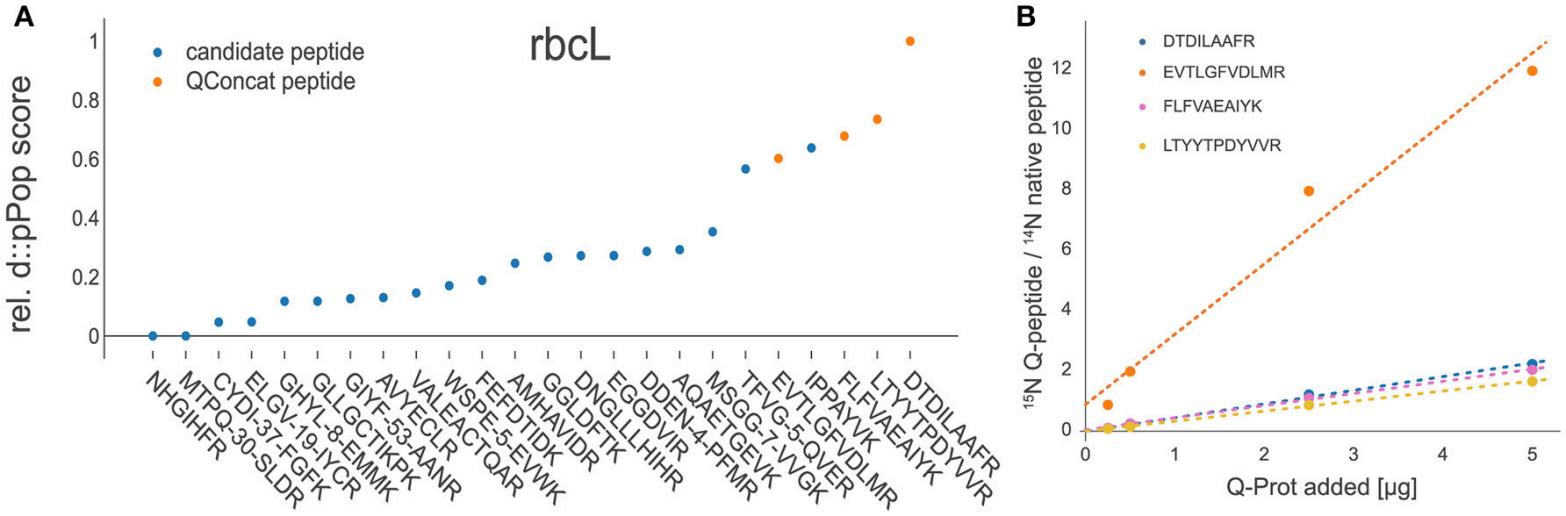
Experimental validation of d::pPop predictions. **(A)** Exemplary comparison between d::pPop prediction results on the rbcL (*C. reinhardtii*) protein query and experimentally validated surrogate peptides (orange dots). **(A)** The d::pPop prediction suggested quantifiable peptides as the top hits. **(B)** The Q-peptide with the lowest d::pPop score showed the biggest deviation from the three other surrogate peptides, pointing to a less accurate quantification information. The complete set of peptides present on the PS-Qprot are presented in Supplemental Figures 2 and 3.

After the validation of the d::pPop observability prediction we tested, whether the prediction can be used for signature peptide selection in a targeted quantification assay. For this purpose, we employed experimental data gained during the absolute quantification of C. reinhardtii photosynthesis proteins using the ^15^N-labeled PS-Qprot (Hammel et al., 2018). For each protein covered on the PS-Qprot, we performed an *in silico* tryptic digestion and a d::pPop observability prediction. We sorted each peptide within every protein according to its predicted rank and compared the top-ranking peptides with the manually selected and validated Q-peptides (Figure 6). For 80% of the Q-peptides that have received a high d::pPop score, a robust quantification of the native peptide was achieved, as judged from a low dispersion estimate of quantification values and positive correlations between labeled QconCat and unlabeled *C. reinhardtii* cell extracts on the peptide and protein levels (Supplemental Figures 2 and 3). The comparison indicates that the d::pPop prediction strongly facilitates the correct selection of suitable surrogate peptides for absolute protein quantification.

### Contribution of physicochemical properties

**Figure 7:**
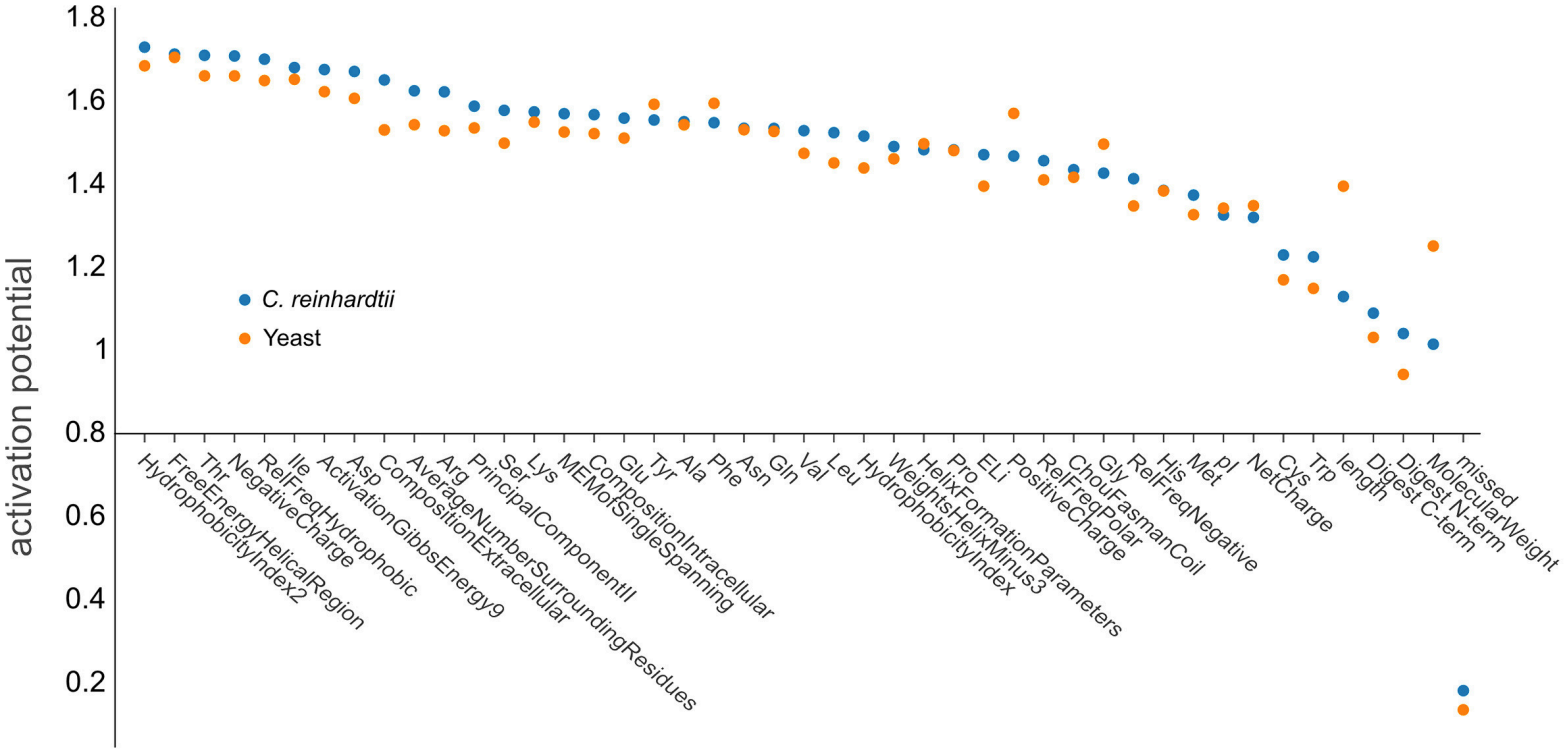
Ranking the influence of physicochemical properties on peptide observability. Net positive contribution of each input dimension (feature) of d::pPops plant (blue dots) and non-plant (orange dots) DNNs in sorted order. The plot shows the different features and their respective activation potential used for learning the models. The learned feature importance differs when learned from yeast (orange) and *C. reinhardtii* (blue) training data.

The use of deep learning neural network approaches enabled us to interrogate our trained artificial network to estimate the importance of individual physicochemical properties for the observability of a given peptide. We followed a procedure according to that described by Roy et al. (2015) to determine the contributions of the neural network nodes to the activation potentials. After the network has been confronted with the training data (76,117 yeast peptides and 76,962 *C. reinhardtii* peptides), the relevance of each individual physicochemical property can be calculated from the different neuronal weights assigned by the network during learning (Figure 7). The small contributions to the activation potential by the different physicochemical properties indicated that PTP prediction is a problem that is not simply explained by a small subset of dominant features, but rather by the complex interaction of more homogeneous features. Especially the impact of structural features has been shown to be uniform, suggesting that microdomain formation, analogous to protein folding, appears to be a common denominator (Weaver, 1982; Islam et al., 2004). It was not surprising that the number of missed cleavages was the feature with the lowest predictive power, as it was included as a negative control since peptides containing missed cleavages were filtered out during the training and prediction steps. However, the analysis revealed that some features are slightly more important than others. Hydrophobicity showed the strongest activation potential, which is in agreement with current literature, explaining the effect by impacts on chromatography and ionization (Jarnuczak et al., 2016). Peptides eluting later in the reversed phase gradient are more hydrophobic and, hence, are likely to ionize better simply due to improved desolvation at the higher organic solvent concentrations required for their elution (Tang and Smith, 2001). Additionally, hydrophobicity may facilitate evaporation from droplets during ESI, because hydrophobic peptides have a higher localization probability toward the droplet's surface (Enke, 1997; Cech and Enke, 2000).

It is also noticeable that the three phosphorylatable amino acids threonine, serine, and tyrosine were under the top 30% of features with a somewhat higher impact on observability. The organism-specific prediction accuracy was consequently manifested by differences in feature importance for both yeast (orange dots) and *C. reinhardtii* (blue dots) proteomes (Figure 7). Substantial differences were apparent regarding the impact of peptide length, the related feature molecular weight, and positive charge. The results further emphasize the need for an organism-specific, or at least organism group-specific, PTP predictor model.

### Web interface d::pPop

**Figure 8:**
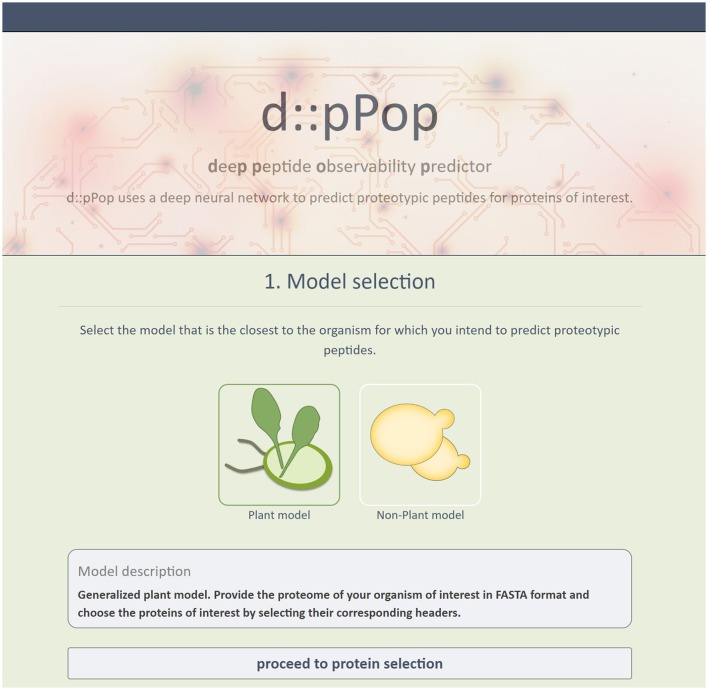
Screenshot of d::pPops Web-Interface. The screenshot shows the implementation of d::pPop as a user-friendly web interface (http://csbweb.bio.uni-kl.de/), which enables researchers to integrate the predictions in their workflow to design targeted protein quantification assays.

In order to use d::pPop to predict PTPs for their own research, users do not have to undergo installation procedures or provide substantial computational resources, but can retrieve ranked PTPs via an easy-to-use web interface (http://csbweb.bio.uni-kl.de/) (Figure 8). The complete procedure is divided into a three-step workflow: (i) Selection of one of d::pPops DNN models, (ii) provision of a protein database in FASTA format, (iii) retreival of ranked peptide sequences. The web interface allows the user to select multiple proteins of interest. This workflow therefore allows for a rapid selection of peptides for targeted proteomics assays based on synthetic peptides or QconCAT proteins.

## Discussion

The prediction of peptides suitable for the absolute quantification of cellular proteins is no easy task. Many labs design targeted assays by selecting peptides based on rules described in the literature (Bereman et al., 2012; Mohammed et al., 2014; Scott et al., 2016). From a global perspective, this results in a quasi-random selection process and leads to a costly experimental trial-and-error method of problem solving. Therefore, any improvement within this workflow can be considered a success. The deep learning-based d::pPop predictor presented in this study proved to be a valuable and robust predictor for peptide observability that performs substantially better than previously described ones. Therefore, it is a valuable method to select candidate signature peptides for targeted protein quantification, especially if MS-based experimental data is fragmentary or missing. In the context of machine learning, the step of selecting positive and negative training data is critical because it is impossible to distinguish between not yet observed (but measurable) and non-observable peptides. In contrast to other predictors like PeptideSieve, CONSequence, and ESPPredictor, our approach circumvents the selection of positive and negative training data sets and exploits the fact that peptides derived from the same protein should all be present at equal amounts in a measurement. Reformulating the prediction problem into a rank regression instead of a binary classification problem might best capture the characteristics of a proteomics workflow as a continuous ranking machine, where differences in ranking are not equidistant. Further, d::pPop predicts PTPs in an organism-specific context that improves the rank prediction as illustrated in Figure 4, and consistently obtains better results compared to other predictors trained on yeast data sets alone (Figure 2). A comparable gain in prediction accuracy in the context of mass and time proteomics has been observed before for the three prokaryotic organisms *Shewanella oneidensis, Salmonella typhimurium*, and *Yersinia pestis* (Webb-Robertson et al., 2008). This suggests that amino acid frequency, composition, and sequence context that vary between organisms, are relevant and need to be modeled to build a useful prediction tool. This is particularly important for plant proteomics and for other research areas, where the availability of shotgun proteomics data is limited. Considering data only from a single study might not provide sufficient information to select peptides that give the desired accuracy in protein quantification.

In this study, we show that all previously published tools perform better than random selection and that our deep rank learning-based approach increases prediction performance significantly (Figures 2, 4). One could argue that the underperformance of ChemScore, PeptideSieve, CONSequence, and ESPPredictor compared with d::pPop is influenced by aspects of the nDCG@4 score used here as evaluation metric. This metric rewards accurate predictions at the top ranks and reduces the punishment for inaccuracies in the lower ranks. We think that this metric reflects very well the process of selecting only few surrogate peptides to target the protein of interest for quantification and is therefore well suited for the comparison of different prediction performances. Moreover, also the PeptideRank algorithm uses the nDCG metric for its performance evaluation. Therefore, the reasons for the discrepancies in prediction accuracy must have another origin. The main difference between d::pPop and the other predictors is that d::pPop predicts the rank more indirectly. While methods inspired by web site ranking perform a pairwise comparison of each web site within each query, we use a deep learning architecture to perform a regression-based estimation followed by a transformation that converts the predicted relative peptide intensities (scores) into ranks. We could show that this method performs well when compared with other methods. However, the absence of a perfect correlation between observed and predicted peptide intensities of the QconCAT protein (Figure 5) indicates that some factors contributing to peptide observability are not captured by d::pPop. Reasons for this might be that the amino acid properties ignore the amino acid sequence context or consider it in a weaker and more indirect manner than covered by the organism specificity. More importantly, even though d::pPop feature selection takes the cleavage efficiency into account, the models to predict cleavage efficiency are helpful but incomplete (Siepen et al., 2007; Fannes et al., 2013; Meyer, 2014). We also neglected to separate effects specific to the measurement device, which might impact the detectability of certain peptides. Nevertheless, it seems that our models trained on the combined data sets from different mass spectrometers predict the observability of the QconCAT peptides measured on a Triple-TOF 6600 (Sciex) instrument reasonably well (Figure 5). At last, an important factor difficult to account for are post-translational modifications (PTMs) that change the relative abundance of a peptide. In cases where the peptide identification search algorithm was not configured to search for specific PTMs, peptides containing PTMs are observed with a lower relative peptide abundance and ranked low. This might be the reason for the higher impact of the phosphorylatable amino acids Ser, Thr, and Tyr on peptide observability when compared with most other amino acids (Figure 7). In future one can include large PTM studies to account for more diverse changes in observability caused by PTMs. The prediction would then depend more on the biological context of the targeted proteomics study, like different environmental conditions or developmental stages. Beyond designing high quality QconCAT proteins using the d::pPop algorithm one can think of other fields of application. Predicted peptide observability could be used to narrow the search space either in classical identification subsequent to a data dependent MS run or prior to a data independent MS run for the design of a spectral library. Further, the d::pPop prediction may potentially aid to increase peptide and protein identification by adding a supplementary feature to discriminate between correct and incorrect peptide sequence assignments. Consequently, it could extend the feature space of popular tools like Percolator (Käll et al., 2007) or MSBayes (Li et al., 2009).

## Conclusion

The prediction of peptide observability is a crucial step to enable researchers to choose suitable surrogate peptides for the design of targeted protein quantification assays. To successfully integrate the d::pPop prediction results into the design, it is recommendable to further prioritize the list of ranked peptides by taking additional aspects into consideration: (i) known PTMs under specific conditions, (ii) digestion efficiency, and (iii) potentially interfering transitions. An accurate prediction of the top peptides and a succeeding, thorough design of targeted proteomics assays considering additional, method-specific requirements is expected to decrease the cost and the labor significantly compared to selecting wrong and weak surrogate peptides for subsequent assay development to perform proteome-wide studies. To facilitate the process, we here release the non-plant and plant-specific rank models to be used by other researchers, implemented in a user-friendly d::pPop web interface (http://csbweb.bio.uni-kl.de/).

## Author contributions

TM and DZ collected and processed the data. DZ designed and implemented the deep learning algorithm. KS implemented the web interface. FS designed the QconCAT protein and performed the LC-MS/MS analyses. MS and TM conceived and supervised the work. TM and DZ wrote the article with contributions from all other authors.

### Conflict of interest statement

The authors declare that the research was conducted in the absence of any commercial or financial relationships that could be construed as a potential conflict of interest.
