# Supplementary material for: Artificial Intelligence Understands Peptide Observability and Assists With Absolute Protein Quantification
Source: Front Plant Sci. 2018 Nov 13;9:1559. doi: 10.3389/fpls.2018.01559 (PMC6242780; doi:10.3389/fpls.2018.01559)
Supplement: Supplementary file 1 [file Presentation_1.PPTX]

## Slide 1
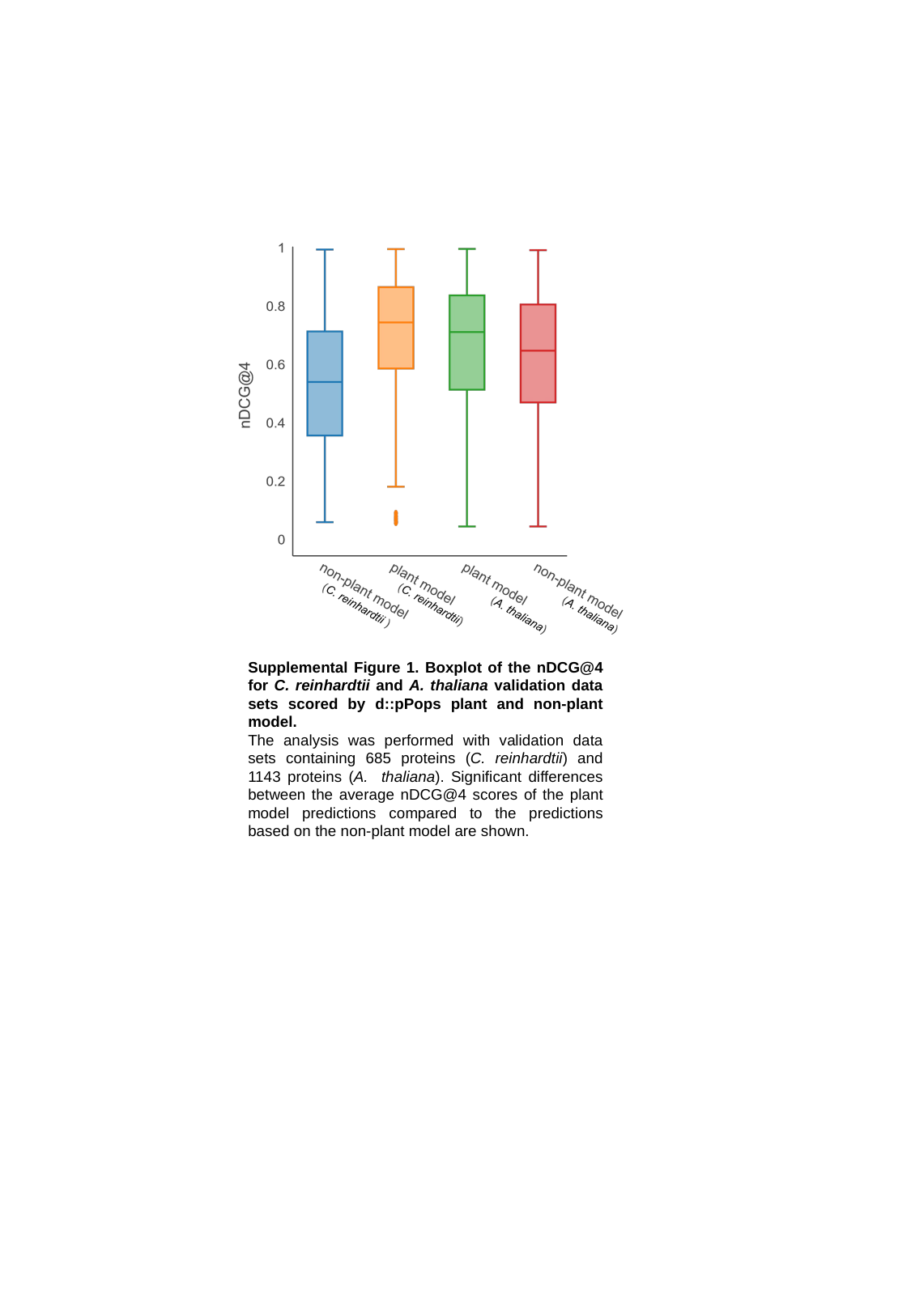

Supplemental Figure 1. Boxplot of the nDCG@4 for C. reinhardtii and A. thaliana validation data sets scored by d::pPops plant and non-plant model.
The analysis was performed with validation data sets containing 685 proteins (C. reinhardtii) and 1143 proteins (A. thaliana). Significant differences between the average nDCG@4 scores of the plant model predictions compared to the predictions based on the non-plant model are shown.
