# Supplementary material for: Artificial Intelligence Understands Peptide Observability and Assists With Absolute Protein Quantification
Source: Front Plant Sci. 2018 Nov 13;9:1559. doi: 10.3389/fpls.2018.01559 (PMC6242780; doi:10.3389/fpls.2018.01559)
Supplement: Supplementary file 2 [file Presentation_2.PPTX]

## Slide 1
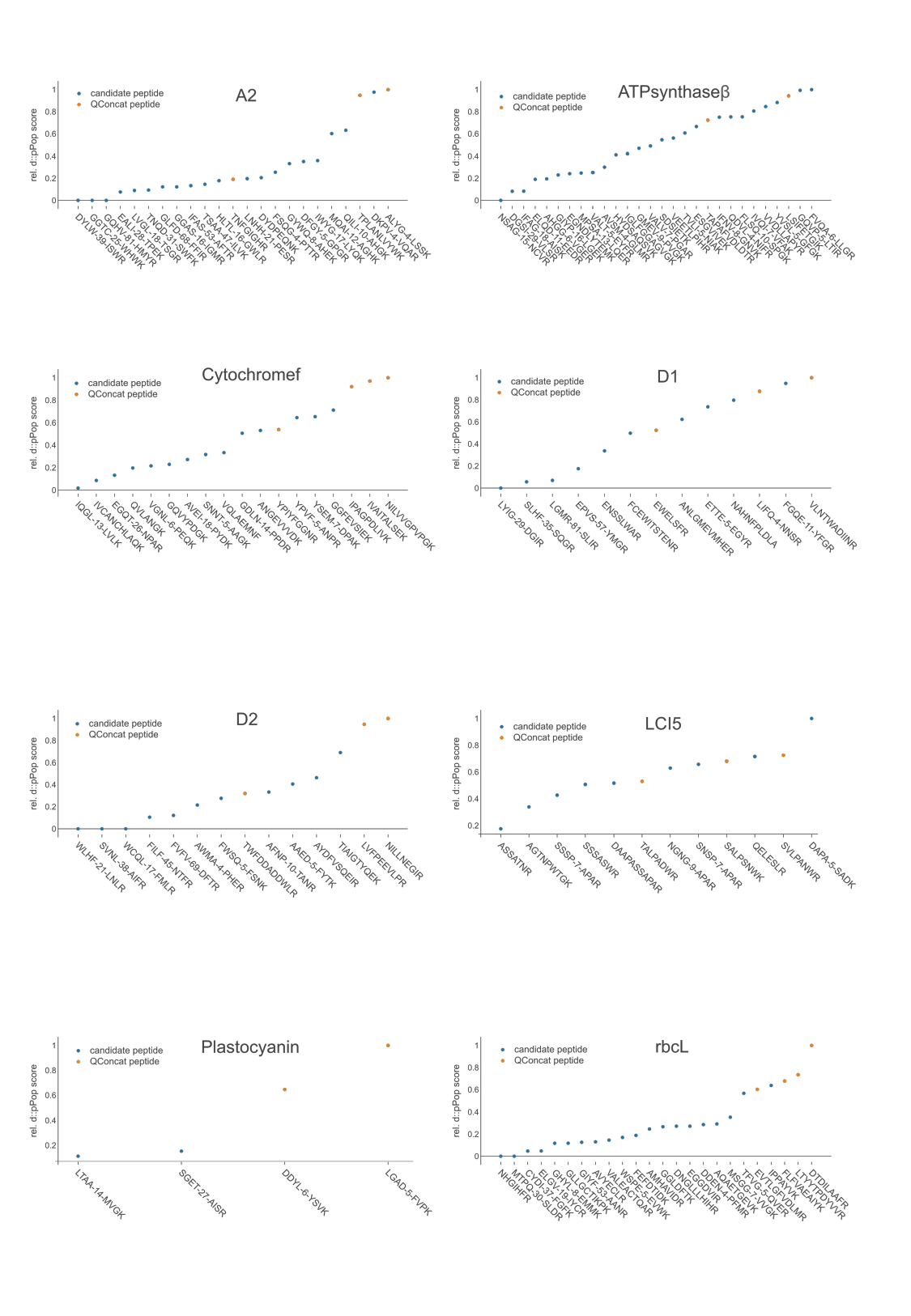

## Slide 2
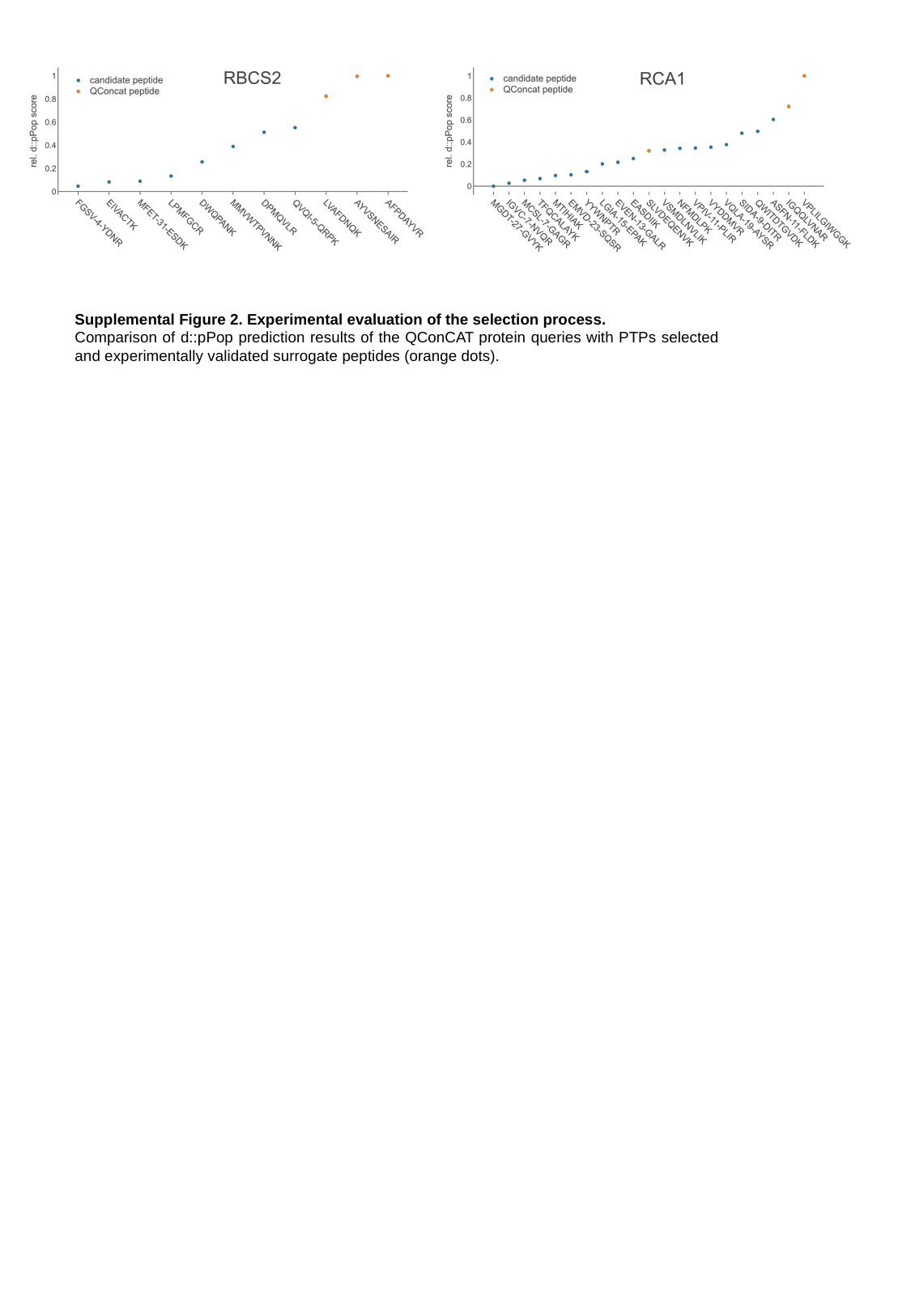

Supplemental Figure 2. Experimental evaluation of the selection process.
Comparison of d::pPop prediction results of the QConCAT protein queries with PTPs selected and experimentally validated surrogate peptides (orange dots).
