# Supplementary material for: Artificial Intelligence Understands Peptide Observability and Assists With Absolute Protein Quantification
Source: Front Plant Sci. 2018 Nov 13;9:1559. doi: 10.3389/fpls.2018.01559 (PMC6242780; doi:10.3389/fpls.2018.01559)
Supplement: Supplementary file 3 [file Presentation_3.PPTX]

## Slide 1
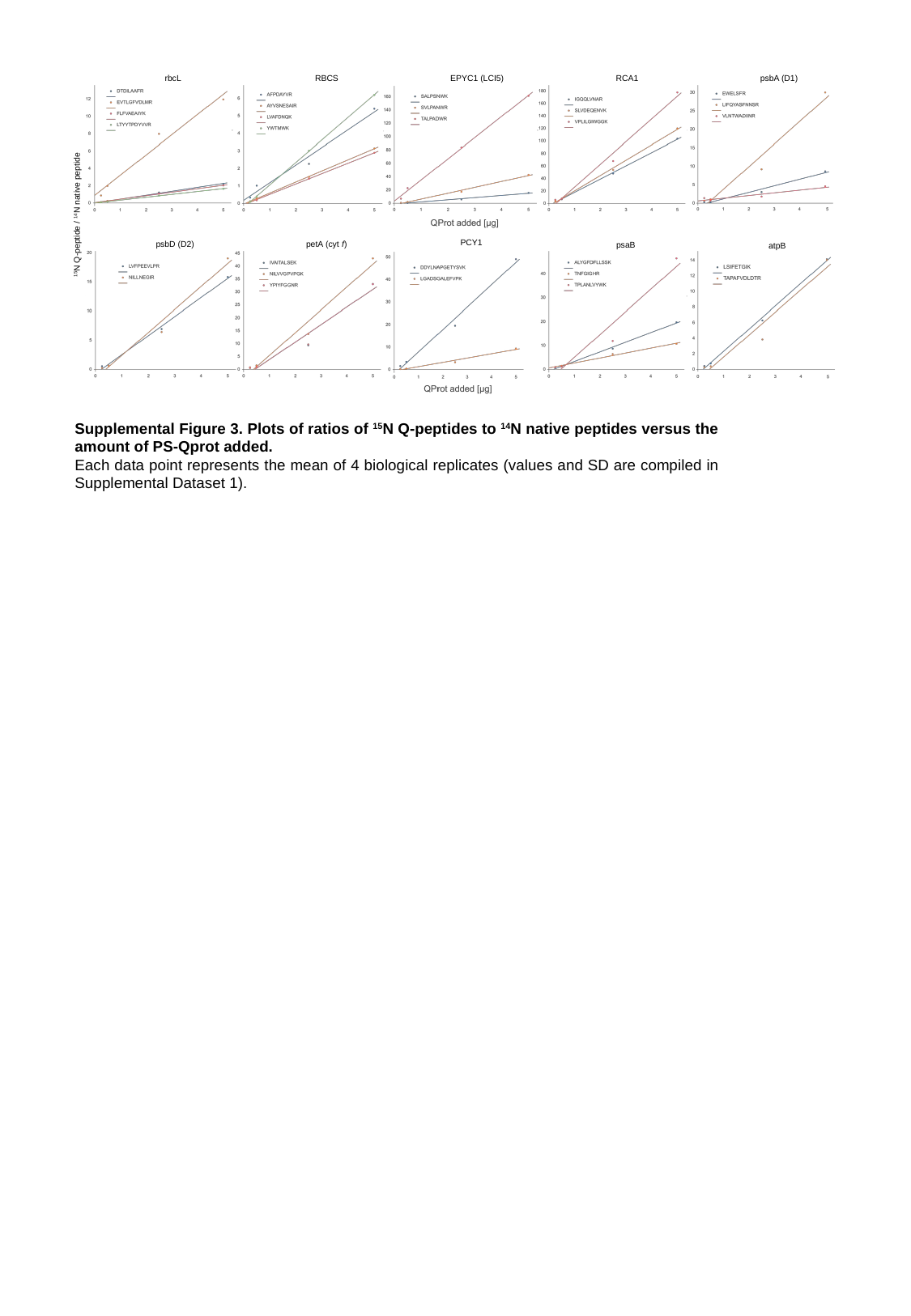

psbA (D1)
RCA1
EPYC1 (LCI5)
rbcL
RBCS
15N Q-peptide / 14N native peptide
PCY1
psbD (D2)
petA (cyt f)
psaB
atpB
Supplemental Figure 3. Plots of ratios of 15N Q-peptides to 14N native peptides versus the amount of PS-Qprot added.
Each data point represents the mean of 4 biological replicates (values and SD are compiled in Supplemental Dataset 1).
